# Depth of Central Venous Catheterization by Intracardiac Electrocardiogram in Adults

**DOI:** 10.5812/aapm.7557

**Published:** 2013-01-01

**Authors:** Prerana N. Shah, Deepa Kane, Jithesh Appukutty

**Affiliations:** 1 Department of Anaesthesiology, Seth GSMC and KEM Hospital, Parel, Mumbai, India

**Keywords:** Heart, Catheterization, Electrocardiography, Veins

## Abstract

**Background:**

Central venous catheterization is done frequently in cardiac surgery and intensive care settings. Faulty positioning of the catheter can cause many complications.

**Objectives:**

The aim of our study was to study the average depth of insertion and formulate a general guideline through the right internal jugular vein (IJV).

**Patients and Methods:**

The right IJV was cannulated over a period of four months and catheter tip positioning was guided by means of an intracardiac electrocardiogram (ECG). Insertion depth was registered at the position of maximum P wave amplitude and the catheter was fixed after withdrawing 2 cm. Pearson’s correlation coefficient was calculated to categorize any relationship between plots of distance versus patient’s height, and regression lines and equations were also calculated. Bland-Altman analysis of data was done to compare the old formulae with our derived formulae.

**Results:**

A total of 155 adult patients were studied. Distances measured were found to be highly correlated with a patient’s height, followed by body surface area (BSA) and weight. For right IJV cannulation in valvular surgeries in adults, the depth of insertion (cm) was (height in cm / 15) + 2 ± 1.58 (SD) and in non-valvular surgeries in adults, it was (height in cm/15) + 1.4 ± 1.47 (SD). The bias was very small when the new formulae were compared to the existing formulae.

**Conclusions:**

The devised formulae predicted the required depth of catheters thereby reducing the possibility of complications and need for radiographic confirmation.

## 1. Background

Placement of a central venous catheter or central venous catheterization (CVC) is a frequent procedure during cardiac anesthesia and intensive care. Its malposition can cause faulty and erroneous central venous pressure measurement along with several fatal complications such as; thrombosis of the great vessels, arrhythmias, cardiac perforation and cardiac tamponade. To avoid potentially fatal complications, it is recommended that the CVC should be placed in the distal superior vena cava (SVC) ie, outside of the cardiac chamber. Malposition related complications could be serious. Thus, in order to suggest a guideline for the proper length of a CVC inserted through the internal jugular vein (IJV), we measured the distance from the skin puncture site to the SVC-right atrium (RA) junction using intracardiac lead ECG monitoring.

## 2. Objectives

The aim of our study was to study the average depth of insertion in the central venous catheter and to formulate a general guideline for the depth of the CVC through the right IJV. The institute's Ethics Committee approval and a valid informed consent were obtained.

## 3. Patients and Methods

Patients were selected according to the inclusion criteria; patients requiring central venous catheterization and having a normal sinus rhythm, and the exclusion criteria being; patient refusal or any abnormal ECG rhythm. Constant and standard approach used for IJV catheterization was the anterior approach (skin puncture at the apex of the triangle formed by the two heads of the sternocleidomastoid muscle). CVC was carried out using the Seldinger technique. A standard, multi-lumen, central venous catheter with a cable and clip was used for connecting the guide wire to a Certodyn® universal adaptor (B. Braun Melsungen, Germany). After identifying the IJV, the guide wire was used as a unipolar electrode. A black marking on the guide wire indicated that the J tip of the guide wire was just at the level of the tip. The sterile connection cable was attached to the guide wire via the clip to the Certodyn® universal adaptor, which allowed switching from a surface to an intracardiac ECG. The CVC was advanced, until the P wave amplitude remained the same after its rise. The right arm electrode (red or RA) of the ECG was connected to the adaptor so that lead II would be showing the intracardiac lead II. Markings on the catheter allow the depth of insertion to be measured. The guide wire was removed and the CVC was withdrawn by 2 cm. The catheter was then fixed to the skin. Intraoperatively, the position of the tip of central venous catheter was confirmed by the operating surgeon, either by palpation of the tip or by visualization if the right atrium was opened. All data were tabulated and analyzed systematically. The data was divided into patients for valvular surgeries and non-valvular surgeries assuming that, as the heart chambers would be enlarged in valvular disease, the depth may vary. All data were expressed as mean ± SD.

### 3.1. Statistical Analysis

The demographic data were analyzed using a Chi-square test. A *P* value < 0.05 was considered significant. Pearson’s correlation coefficient was calculated to categorize any relationship between; height, weight or body surface area (BSA) and the depth of insertion required to locate correct positioning of central venous catheter. Plots of distance were made and regression lines and equations were calculated by using SPSS 14.0 (SPSS, Chicago, IL). Bland-Altman analysis of data was conducted to compare the existing formulae with our derived formulae.

## 4. Results

**Figure 1.**
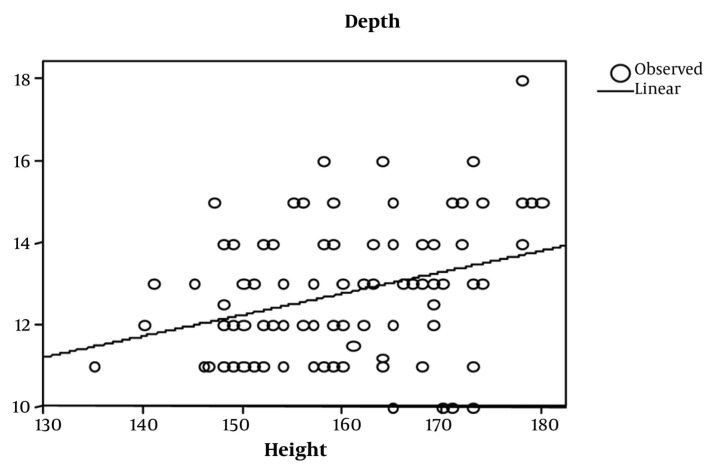
Depth vs. Height in Adult Valves (r ^2^ = 0.101, P = 0.001)

**Figure 2.**
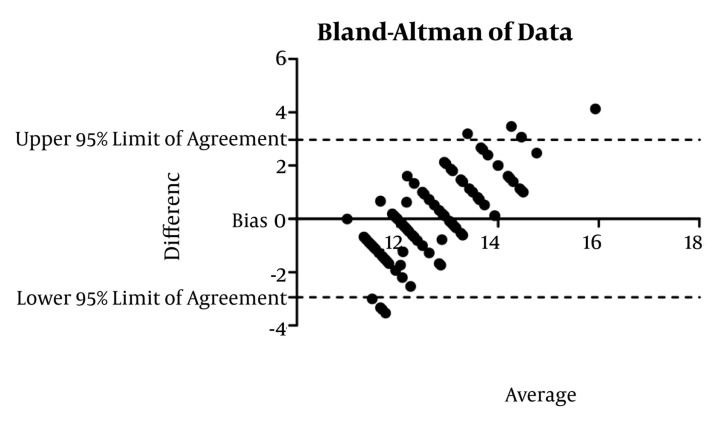
Bland Altman of Data, Adult Valvular Disease

**Figure 3.**
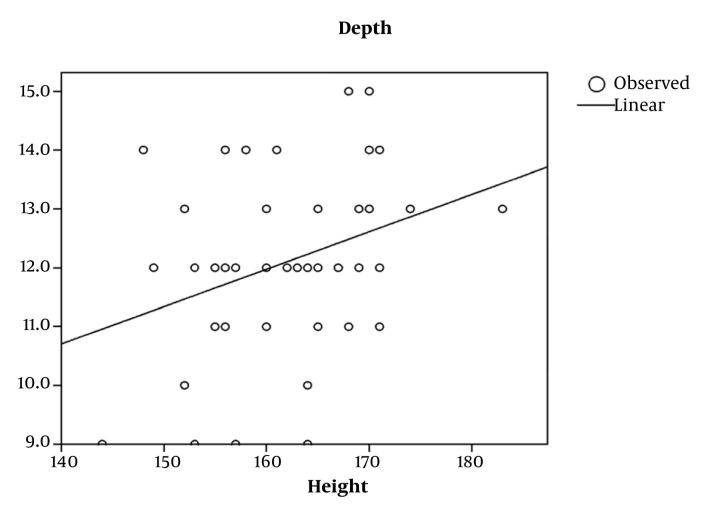
Depth vs. Height in Adult Non-Valvular Surgical Patients (r ^2^ = 0.118, P = 0.016)

**Figure 4.**
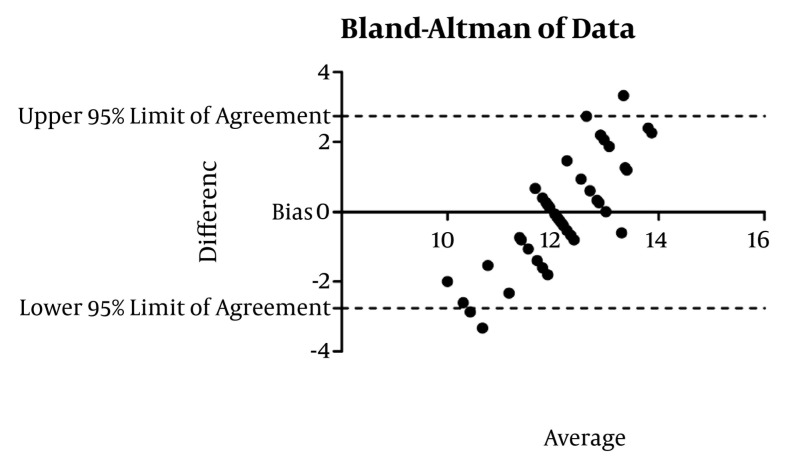
Bland Altman of Data, Adult Non-Valvular Disease

CVC was studied in 155 adult patients. There was no significant gender based differences among the different groups (*P* = 0.087) (Table 1). In the adult, valvular heart disease group, depth had the best correlation with BSA (r = 0.356 *P* < 0.001), followed by weight and height. In the adult (non-valvular) group, height correlated best with depth (r = 0.343 *P* = 0.016). The depth of insertion was significantly greater in patients undergoing valve surgeries (*P* = 0.0149) compared to adults in the other group (Table 2). Overall height had a high correlation (r = 0.875 *P* < 0.001) with depth of insertion followed by BSA (r = 0.867 *P* = 0.000) and weight (r = 0.817 *P* = 0.000). Height had a significant correlation in all of the groups, so further analysis was restricted to height in each group. Simple formulae were developed to predict placement of CVC through the right IJV. These were developed by plotting a straight line on the graph of a patient’s height versus depth and then a simple formula describing that line was derived (Figures 1 and 3). For right IJV cannulation of adults (valves): depth of insertion (cm) = (height in cm / 15) + 2 ± 1.58 (SD). Adult (non-valvular): depth of insertion (cm) = (height in cm/15) + 1.4 ± 1.47 (SD) After applying Bland-Altman to the data in adult patients undergoing valvular surgery, the bias (ie, average difference between the two sets of readings) was small with the new formula (depth measured with the new formula was 0.018 less compared with the original) with a SD of 1.50. In adults for non-valvular surgery, the bias was small with the new formula (depth measured with the new formula was 0.0100 units more compared with the original) with an SD of 1.40 (Figures 2 and 4). The width of 95% confidence interval with the new formula was less compared with the width of the 95% confidence interval with the old formula.

**Table 1.** Demographic Data

Adult	Male, No	Female, No.	Total, No.
**Valves**	58	44	102
**Non-Valvular**	33	20	53
**Total, No.**	138	86	224

**Table 2.** Depth of Central Venous Catheterization

	Mean ± SD	r ^c^	Range	*P* value
**Adult Valves, n = 102**				
Weight, kg	49.30 ± 11.66	0.36 b	30 - 85	0.001
Height, cm	159.82 ± 10.07	0.32 b	135 - 180	0.002
Bovine Serum Albumin	1.47 ± 0.20	0.37 b	1.06 - 2.05	0.000
Depth, cm	12.67 ± 1.58			
**Adult Others, n = 53**				
Weight, kg	57.26 ± 16.51	0.36	30 - 85	0.059
Height, cm	162.26 ± 7.78	0.30 a	144 - 183	0.016
Bovine Serum Albumin	1.59 ± 0.24	0.36 a	1.12 - 1.96	0.044
Depth, cm	12.21 ± 1.47			

^a^Correlation is significant at 0.05 level (2-tailed).

^b^Correlation is significant at 0.01 level (2-tailed).

^c^r Pearson’s correlation coefficient of depth with weight, height and body surface area.

## 5. Discussion

It is recommended that the CVC should be placed in the distal superior vena cava (SVC) ie, outside of the cardiac chamber. Incorrect positioning can be prevented by; correctly judging catheter length, using a gentle technique when advancing the catheter, and positioning the patient in a way that facilitates access to the vein. Studies conducted over the past several years have proven that the use of an intracardiac ECG may obviate the need for routine x-rays to verify CVC placement. In 1949, Hellerstein took his previous experience with ECG leads and applied this in a unique way for directly recording atrial potentials. He used a CVC filled with saline solution. The signal was captured at the catheter hub through a self-made adaptor and then transferred to an ECG monitor. In his landmark publication, he examined the recording of both the saline solution and through a metal wire. As soon as the catheter tip reaches the sinoatrial node, there is an abrupt increase in the height of the P wave (1, 2). When the tip enters into the RA, the height of the P wave potential is reached. A disposable electrode is connected to a universal adaptor, which can be switched back and forth between the skin and intra-arterial leads. The intracardiac ECG lead produces accurate information about catheter tip position for all patients, except for those with pre-existing abnormal cardiac rhythms. Numerous clinical studies have demonstrated a high accuracy rate of over 90% for this method. However, in our population, the distance does not correspond according to any of the following published rules of thumb: right subclavian or jugular vein, 13-16 cm; left subclavian or jugular vein, 15-20 cm^2^.

Peres et al. published catheterization guidelines for adult patients (3). Peres devised the formula for access through the subclavian and jugular vein, while there was insufficient data available for other puncture sites. The following relationship was established, right side; external and internal jugular vein, L = H/10. The values obtained using the Andropoulos formula contradicts those of the Peres formula in patients taller than 100 cm, and when access is made through the jugular vein (3, 4). This discrepancy merits further investigation, especially in our population, hence, our study was designed. In patients with dysrhythmia, atrial fibrillation or implanted pacemakers, the P wave remains unchanged in the intra-arterial ECG lead; even if the catheter is correctly withdrawn from the atrium into the vena cava. A single report of catheterization of the carotid artery with advancement into the right atrium has been made, which resulted in the induction of P wave elevation. In this case, the ECG signal was interpreted as confirming the correct placement, however, the catheter had been seriously misplaced (5). Constant position monitoring is required to instantly identify problems that might arise during puncture or advancement (6). Atrial ECG recording is therefore recommendable, not only for economic reasons, but because of its high success rate. The use of an intracardiac ECG is an easy method, which can be conducted at minimal cost (7). It helps to detect atrial placement in each patient individually and reduces the impact of anatomical differences among patients of the same height and weight that can give rise to different distances between the insertion point and atrium and SCV-atrial junction. The use of an ECG lead to verify CVC position and ultrasound has now become a standard technique in anesthesia and intensive care medicine (8). Physicians who have mastered these techniques therefore no longer need to perform chest x-rays to monitor catheter position, since the ECG lead provides sufficiently accurate verification. The devised formulae can reasonably predict the required depth required for central venous catheters, thereby reducing the possibility of complications, need for radiographic confirmation and it also raises safety levels (8).
